# Editorial: Changing backgrounds and groundbreaking changes: gynecological surgery in the third decade of the 21st century volume II

**DOI:** 10.3389/fsurg.2025.1587048

**Published:** 2025-03-19

**Authors:** Rafał Watrowski, Radmila Sparić

**Affiliations:** ^1^ Department of Gynecology, Helios Hospital Müllheim, Müllheim, Germany; ^2^ Faculty of Medicine, University of Freiburg, Freiburg, Germany; ^3^ Clinic for Gynecology and Obstetrics, University Clinical Centre of Serbia, Belgrade, Serbia; ^4^ Faculty of Medicine, University of Belgrade, Belgrade, Serbia

**Keywords:** gynecologic surgery, minimally invasive surgery, laparoscopy, robotic surgery, cervical cancer, rare tumors in gynecology, pelvic organe prolapse (POP), fertility preservation in cancer patients

Editorial on the Research Topic Changing backgrounds and groundbreaking changes: gynecological surgery in the third decade of the 21st century volume II

The first volume of this Research Topic (RT) focused largely on patient safety and complication management (1). In this second volume, the submitted manuscripts also group around key contemporary themes. For instance, four papers are dedicated to the robotic approach (Ferrari et al., Kawamura et al., Neis et al., Ascione et al.), while four deal with cervical neoplasia (Ning et al., Zhang et al., Zeng et al., Li et al.). Among these, two evaluate a de-escalation of the surgical approach (Ning et al., Zeng et al.).

We believe that this RT accurately reflects the current discussions and evidence gaps in gynecologic surgery. In 2017, the LACC trial reshaped gynecologic oncology by demonstrating that laparoscopic radical hysterectomy (RH) for cervical cancer (CC) compromises treatment outcomes compared to the open approach (2). These findings were recently confirmed in the final overall survival analysis (3). Further analyses of the same dataset showed no difference in complication rates between open and laparoscopic RH (4), reaffirming the open approach as the standard of care for CC after two decades of laparoscopic RH evolution (3). Recently, the SHAPE trial demonstrated comparable oncological outcomes between simple hysterectomy (SH) and radical hysterectomy (RH) for early-stage, low-risk cervical cancer, confirming that surgical de-escalation can be considered safe in such cases (5). This RT includes a closely related meta-analysis by Zeng et al., examining the efficacy and safety of non-radical surgery for early-stage CC. The “groundbreaking changes” in CC surgery are accompanied by evolving anatomical knowledge, prompting a reassessment of the current anatomical classification of RH (6). In the coming years, the role of robotic approaches in CC treatment will be clarified by the RACC trial (estimated completion: May 2027) (7). Additionally, the newly launched LASH trial will address both surgical de-escalation and the relevance of surgical approach (8).

A decade ago, evaluations of robotic-assisted approaches primarily focused on feasibility, safety, and cost compared to conventional laparoscopy (9). These evaluations typically demonstrated similar complication rates and surgical outcomes, while favoring robotics for improved dexterity, visibility, and surgeon comfort. However, higher costs remained a limiting factor, particularly in low-volume hospitals and resource-limited settings (9). Moreover, robotic-assisted surgery was almost exclusively associated with the pioneering da Vinci platform and its subsequent generations. Today, the market offers more than twenty robotic platforms, including various da Vinci variants (Intuitive Surgical Inc., California, USA), Senhance® (Asensus Surgical, North Carolina, USA), Versius (Cambridge Medical Robotics, UK), and the Hugo™ RAS system (Medtronic, Minneapolis, USA) (10, 11).

Technical advancements in robotic surgery have been applied early in endometriosis surgery, which is often characterized by extreme complexity and the necessity of nerve and fertility preservation. The review by Ferrari et al. addresses the role of robotic surgery in deep-infiltrating endometriosis, separately analyzing critical localizations such as colorectal, diaphragmatic, and sacral plexus endometriosis. Beyond summarizing current evidence, the authors highlight gaps in knowledge and emphasize the need for prospective randomized controlled trials. Kawamura et al. evaluated the feasibility of omitting a uterine manipulator during robotic-assisted hysterectomy without compromising patient safety. Their conclusion suggests that, unlike conventional laparoscopic hysterectomy—where a uterine manipulator is usually indispensable—the precision of robotic systems may reduce the necessity for a manipulator in certain cases. However, a “difficult” surgical field (e.g., ovarian casts or Douglas obliteration) and higher patient BMI still necessitate its use. In such cases, the employment of a fourth robotic arm could enhance surgical independence and resource efficiency. A surgeon's impact on patient safety is significantly influenced by surgical training, case volume, and various factors encompassed by the “human factor”, including individual health, personality, and workload (9). Neis et al. used visualization techniques to analyze workflow consistencies and variabilities among surgeons performing robotic total laparoscopic hysterectomy, applying objective measurements to assess individual surgical behavior.

The collection of papers dedicated to robotic approaches is rounded out by the work of Ascione et al., which describes how the robotic-assisted approach can enhance fertility-preserving treatment of cornual pregnancy. This is the second paper in this RT addressing fertility-preserving approaches. Fertility-sparing surgery for early-stage CC patients is of great importance given the trend of childbearing shifting into the third and fourth decade of women's lives. The evaluation of clinicopathological characteristics by Ning et al., based on a large cohort of 10,629 stage I CC patients aged 15–39 years, provides valuable insights into fertility-sparing decision-making and represents an important contribution to this RT.

The work of Malanowska-Jarema et al. continues the evaluation of laparoscopic lateral suspension (LLS), which was suggested in Volume 1 of this RT as the new gold standard for treating pelvic organ prolapse (POP) (12). Their work provides evidence of the equivalence of LLS to laparoscopic sacrocolpopexy in terms of sexual function. This is a valuable contribution, as a contemporary “standard of care” for POP can only be established by evaluating a broad spectrum of outcomes.

An important part of this RT consists of carefully selected case reports. It is commendable that the journal still values case reports on par with studies with higher citation potential. Many journals have banned case reports in response to competitive pressures to optimize citation metrics and impact factors, as these productivity metrics (despite ongoing critiques) remain central to both academic careers and journal reputations (13). Notably, bibliometric studies have now evolved into an independent research field, as seen in the paper of Pérez-Reátegui et al. (14). However, without case reports, building a stable body of evidence for rare diseases would be nearly impossible (15). Two exemplary case reports in this RT focus on cervical tumors: one describes a rare ureteric-bud adenocarcinoma misdiagnosed as a cervical fibroid (Zhang et al.), while the other reports on benign cervical malakoplakia confused with CC (Li et al.). These cases underscore the continued importance of case reports, as demonstrated here in CC, since a small fraction of clinical presentations will always fall outside established frameworks, requiring an intuitive approach or treatment based on analogy to existing pathways (16).

To look forward, we predict that the renaissance of robotic surgery is occurring now, marking a shift from “robotic-assisted” to “robotic-guided” surgery through the implementation of artificial intelligence to integrate augmented reality and multimodal information (including imaging techniques, radiomics, and molecular diagnostics) into a virtually enhanced surgical field. These advancements will set new milestones in surgical approaches and personalized patient care. In the coming years, we anticipate further refinements in the surgical management of CC, informed by ongoing trials, as well as continued evolution in endometrial cancer treatment through molecular classifications and the establishment of sentinel node biopsy as the standard of care. We hope that, in rare diseases, continuous publication of case series—along with improved publication standards and integration of molecular analytics—will allow for reliable synthesis and cautious standardization of treatment approaches, including fertility-sparing criteria for rare malignancies (17).

We thank all authors who contributed to this issue and hope that their publications will contribute to and inspire further “groundbreaking changes” and “changing backgrounds” in gynecological surgery.
